# Seroprevalence of transfusion transmitted infections among blood donors in Gash Barka Zonal Blood Transfusion Center, Barentu, Eritrea, 2014 through 2017

**DOI:** 10.1186/s12878-019-0136-5

**Published:** 2019-03-12

**Authors:** Yacob Tesfamichael Keleta, Oliver Okoth Achila, Absera Woldu Haile, Bereket Habteslasie Gebrecherkos, Danait Tareke Tesfaldet, Kibrom Solomon Teklu, Mesuda Abrhum Mohammed, Selihom Tesfaslase Ghedel

**Affiliations:** 1 Department of Clinical Laboratory Science, Asmara College of Health Sciences, P.O. Box 8566, Asmara, Eritrea; 2 Department of Biomedical Sciences, Asmara College of Health Sciences, Asmara, Eritrea

**Keywords:** Hepatitis B virus, Human immunodeficiency virus, Hepatitis C virus, Syphilis, blood donation

## Abstract

**Background:**

Transfusion-transmissible infections pose a major health risk in developing countries, including Eritrea. In the present study, we sought to determine the prevalence of specific transfusion transmitted infections (TTIs) and the associated risk factors among blood donors at a newly established regional blood transfusion center in Barentu, Eritrea.

**Methods:**

The seroprevalence of markers for specific TTIs by sex, age, educational status, residence, occupation, and donor type was evaluated for donors who donated blood between July 2014 and April 2017. The relationship between TTIs and the stated factors was evaluated using the Pearson Chi-square test/Fishers exact test. Adjusted and unadjusted binary logistic regression models were employed to estimate the odds ratio (OR) and 95% confidence interval (CI) for the occurrence of TTIs. A two-sided *p*-value < 0.05 was considered statistically significant.

**Result:**

A total of 1939 donors were included in this study. Majority of the donors were males (88.2%), urban residents (68.8%), greater than 25 years of age (67%), and family replacement blood donors (FRBD) (59.7%). Two hundred and fifty (12.9%) donors were infected by at least one TTI. The cumulative seroprevalence of Human immunodeficiency virus, Hepatitis B virus, Hepatitis C virus and syphilis were 16 (0.8%), 97 (5%), 13 (0.7%) and 140 (7.2%), respectively. Out of the total 266 infected donors, the prevalence of co-infection was 16 (0.8%). In the adjusted model, the OR and 95% CI for the seropositivity for any TTI associated with age, no formal education, elementary school educational level, and junior school educational level were 1.02 (95% CI: 1.01–1.04), 4.4 (95% CI: 2.58–7.49), 2.67 (95% CI: 1.49–4.80), and 2.00 (95% CI: 1.14–3.52), respectively. In addition, blood from FRBD had an increased likelihood of contamination with at least one TTI, with an OR (95% CI) of 1.56 (1.10–2.21).

**Conclusion:**

The prevalence of transfusion-transmissible infections is relatively high. In particular, specific groups in the population appear to be disproportionally affected. Therefore, targeted sensitization campaigns should be implemented in the future.

## Background

Blood transfusion and component therapies are integral part of modern medical interventions. However, these therapies are not risk free with risk ranging from potentially fatal immuno-hypersensitivity reactions to potential transmission of blood-borne pathogens from donor to recipient. According to some reports, there is an estimated 1% likelihood of transfusion-linked risk in some developing countries [1]. Human immunodeficiency virus (HIV) 1 and 2, hepatitis B virus (HBV), hepatitis C virus (HCV) and *Treponema pallidum* (the etiological agent for syphilis) contribute to a significant proportion of the Transfusion-transmissible infections (TTIs) [2].

To limit the risk associated with TTI exposure, the World Health Organization (WHO) recommends mandatory screening of all blood donors and units for HIV 1 and 2, HCV, HBV and syphilis infections [3]. These recommendations combined with the development of newer and more sensitive screening tests have significantly reduced the incidence and prevalence of TTIs in countries where such approaches have been implemented [4, 5]. Nevertheless, TTI risk remains relatively high in low-income countries compared with high-income countries [4]. A multicenter collaborative study on the risk of HIV transmission through blood transfusion in Sub-Saharan Africa (SSA) reported that the risk of HIV varied from 1 in 25,600 in Congo to 1 in 90,200 in Senegal [6]. The TTI risk remains high in SSA despite the support of transfusion safety received by the WHO and other organisations [2].

The limitations associated with commonly used viral detection techniques, such as over-reliance on rapid test-based screening/serological test kits contribute to a substantial risk of TTI. The donors in the ‘window period’ or donors in a ‘low level carrier state’ (when markers of chronic infection are below the sensitivity of existing assays) pose a significant degree of risk [7]. A survey on international quality control challenged the reliance on rapid test-based screening protocols in resource poor settings, as it underscores the test outcome [8]. Other factors for the risk of TTI may include the high prevalence of specific TTIs in the general population, reliance on family replacement blood donors (FRBD), sub-optimal implementation of the WHO guidelines, inadequate screening facilities and surveillance systems, lack of reference screening tests like Enzyme Immunoassays (EIA) or Nucleic Acid Testing (NAT). The lack of routine testing for some infectious agents endemic to SSA has been reported in several studies [2, 9, 10]. Human T cell Lymphotropic Virus (HTLV) 1 and 2, Cytomegalovirus (CMV), Yellow Fever, and Rift Valley Fever are among others. Another unappreciated transfusion risk in SSA involves septic transfusion reactions due to bacterial contamination during collection and processing [10].

The prevailing situation calls for continuous risk assessment and profiling of donors. The availability of robust epidemiological data may help in the identification of TTI trends and associated risks. The information can also be leveraged for donor selection and recruitment [10]. Although several studies on seroprevalence of TTI markers among donors have been undertaken in several countries in SSA [11–14], data on the seroprevalence of major TTIs among blood donors in some countries is incomplete or entirely lacking. For instance, after an extensive search of published literature, we could only locate one study on TTI’s in Eritrea that was conducted at the National Blood Transfusion Center (NBTC) in 2011 [15]. However, the previous study failed to present data on the seroprevalence of major TTIs among blood donors in the various zones of the country. Furthermore, to the extent of our knowledge, there is no any study that explore the relationship between TTIs and a range of critical demographic variables. Therefore, the primary objective of this study was to generate a preliminary report on the prevalence of major TTIs at the recently established Gash Barka Zonal Blood Transfusion Center in Barentu, Eritrea. We explored the relationship between demographic variables and the frequency of hepatitis B virus surface antigen (HBsAg), anti-HCV, anti-HIV, and anti-TP (a marker for syphilis infection) among blood donors.

## Methodology

### Study design and setting

Between July 2014 and April 2017, a retrospective evaluation of all blood donations at the Gash Barka Zonal Blood Transfusion Centre, Barentu, Eritrea was undertaken. Barentu, the Capital city of Gash Barka, is located approximately 237 km to the west of Asmara, Eritrea. The blood bank was established in July 2014 as part of the Eritrean Government’s effort to de-centralize blood processing services. The blood bank provides services for hospitals included in Gash-Barka administration region, namely Barentu, Teseney, Akordet, and Barentu military hospitals. As part of routine practice, all blood donors at the center undergo systematic pre-donation counseling and evaluation. The blood bank regularly records the demographic and clinical data, and previous history of donation in the donors’ register.

### Study population

The present study included 1939 donors. The blood donations were from both voluntary non-remunerated blood donors (VNRBD) and FRBD. Donor selection was based on NBTC approved protocols. Blood donors who were physically fit, aged between 16 and 65 years, and had a body weight greater than 50 Kg were included in the present study. Whereas, blood donors who were anaemic (hemoglobin level: ≤12.5g/dl for females and ≤ 13.5g/dl for males), had recent history or currently diagnosed with jaundice, had recent history of chronic disease, venereal disease, asthma, surgery, high risk behaviour (such as unsafe intercourse), had history of pregnancy and apparently unhealthy or malnourished individuals were excluded.

### Data collection

Data were retrieved from the anonymized blood donors register by the principal investigators with the help of blood bank staff. Donor’s records containing specific demographic information (age, sex, occupation, educational status, residence, sub-zone and ethnicity); the number of donations and the sero-status of the TTI’s were transferred to Excel Spreadsheet (Microsoft Inc.). Donors who visited the blood bank twice or more were identified, and their first visit was considered with the aim to control bias by indication.

### Serology

The center employs two different rapid testing procedures for HIV post-donation screening. The rapid test kits employed for HIV detection during the study period were Determine HIV-1/2 (Abbott Laboratories, Illinois, USA) and Uni-Gold HIV-1/2 (Trinity Biotech, Dublin, Ireland). In line with existing national testing algorithm, positive results were re-tested using EIA. ELISA tests were based on AccuDiag™ HIV 1 and 2 Ag/Ab (Diagnostic Automation/Cortez Diagnostic, California, USA). The SD HBsAG ELISA 3.0, SD Standard Diagnostic, Inc., (Republic of Korea) and HCV ELISA 3.0, SD Standard Diagnostic, Inc., (Republic of Korea) were used to test HBsAg and HCV, respectively. Anti-TP was detected using Rapid Cassette Syphilis Ab test, Mediff Biotech. All the kits are approved by the WHO.

### Data analysis

Data extracted from the institution’s donor records were entered, cleaned and analyzed using IBM SPSS Statistics 20.0 (Armonk, New York, USA). The seroprevalence of TTI was determined by dividing the number of positive cases by the total number of blood donations per year. Total TTI’s, anti-HIV, HBsAg, anti-HCV, and anti-TP frequencies were expressed in percentages. Comparisons for different demographic characteristics and donation frequencies were performed using Pearson Chi-square (χ^2^) test or Fisher’s exact test. Adjusted and unadjusted binary logistic regression models were employed to estimate the odds ratio (OR) and 95% confidence interval (CI) for the occurrence of TTI. A two-sided *p*-value < 0.05 was considered statistically significant.

### Ethics approval

The study protocol was approved by the Ethics Committee of the Asmara College of Health Sciences and the Ministry of Health of the state of Eritrea. Consent to participate in this study was not obtained from the donors since donation records were anonymized via de-identification.

## Results

### Demographic characteristics of blood donors

A total of 1939 blood donors (1710 (88.2%) males and 229 (11.8%) females) were included. The number of donors screened per year and their demographic characteristics are presented in Table 1. The mean age of donors was 32.37 ± 11.23 years (age range = 54 years). Individuals in the age category 36–50 years comprised 33.3% of the donors. Among the donors, 67.4% were from the Tigrigna ethnic group, 53.4% had high school/tertiary level level of education and 59.7% were FRBD.

**Table 1:** Socio-demographic characteristics of blood donors at Gash Barka Zonal Blood Transfusion Center, Barentu (*n* = 1939)

Variables	Categories	Frequency	Percentage (**%**)
Year	2014	286	14.7
	2015	879	45.3
	2016	569	29.3
	2017	203	10.5
Age Category	< 18	258	13.3
	19–25	383	19.8
	26–35	529	27.3
	36–50	645	33.3
	> 50	124	6.4
Sex	Male	1710	88.2
	Female	229	11.8
Ethnicity	Tigrigna	1307	67.4
	Tigre	229	11.8
	Kunama	129	6.7
	Nara	192	9.9
	Others	82	4.4
Residence	Urban	1335	68.8
	Rural	604	31.2
Occupation	Office worker	432	22.4
	House wife	68	35.3
	Student	399	9.0
	Daily worker	684	35.3
	Unemployed	17	0.9
	Others	339	20.6
Educational Level	No education	344	17.7
	Elementary	220	11.3
	Junior	339	17.5
	High school	683	35.2
	Tertiary	353	18.2
Types of Donor	FRBD	1158	59.7
	VNRBD	781	40.3

*VNRBD* Voluntary non-remunerated blood donors, *FRBD* Family replacement blood donors

### Major TTIs

The proportion of samples which were tested positive for at least one TTI was 250 (12.9%). The overall seroprevalence of markers for HIV, HBV, HCV, and syphilis were 16 (0.8%); 97 (5%); 13 (0.7%) and 140 (7.2%), respectively. The highest cumulative seroprevalence proportion (15.3%) was reported in the year 2015. Data for specific TTI is shown in Table 2.

**Table 2:** Year wise infected cases of HIV, HBV, HCV and syphilis from July 2014 to April 2017 at Gash Barka Zonal Blood Transfusion Center, Barentu (*n* = 1939)

Year	Total Tested	HBsAg + ve	HCV + ve	HIV + ve	Syphilis + ve	Any TTI + ve
2014	286	13 (4.5%)	0 (0.0%)	5 (1.7%)	17 (5.9%)	34 (11.9%)
2015	880	60 (6.8%)	8 (0.9%)	7 (0.8%)	69 (7.8%)	135 (15.3%)
2016	569	14 (2.5%)	5 (0.9%)	4 (0.7%)	50 (8.8%)	67 (11.8%)
2017	204	10 (4.9%)	0 (0.0%)	0 (0.0%)	4 (2.0%)	14 (6.9%)
*P*-value		0.003	0.219	0.188	0.008	0.007
Total	1939	97 (5.0%)	13 (0.7%)	16 (0.8%)	140 (7.2%)	250 (12.9%)

Note: *P*-value, Pearson Chi-square test to test statistical difference; *+ ve* positive; *Any TTI* Blood units positive to any of the tested transfusion transmissible infections

### Co-infections among blood donors

In the present study, 16 (0.8%) of the 266 infected donors were co-infected with two pathogenic agents. The proportion of HBV-Syphilis co-infections was 10 (62.5%); HIV-Syphilis 3 (18.75%), HIV-HCV 2 (12.5%), and HCV-Syphilis 1 (6.25%).

### Comparison of serologic markers for specific TTIs based on sociodemographic variables

The demographic characteristics of donors positive for at least one TTI are presented in Table 3. The overall seropositivity rate had a statistically significant difference in gender, age, educational level, occupation, and residence. In particular, the cumulative seroprevalence of TTIs was significantly higher in males compared with females (13.7% vs 7%; *P* = 0.000). Donors aged > 45 years had a higher prevalence of TTIs compared with donors in other age groups (*P* = 0.000). A significantly higher positivity rate was also observed in donors with no formal education 93 (27%), elementary education 41 (18.6%) and junior level education 41 (12.1%) compared to donors with higher level of education (*P* = 0.000). Similarly, this study reported higher seropositivity rate among daily workers 146 (21.3%) and rural dwellers 121 (20%) compared to their respective counterparts (*P* < 0.000). However, there was no statistically significant difference in demographic variables in the prevalence of HBV and HCV seropositivity. Besides, rural dwellers had a higher HIV positivity rate compared to urban residents (1.7% vs 0.4%; *P* = 0.009). A significant difference between occupation and HIV positivity was also observed (*P* = 0.026). The seroprevalence for markers of syphilis showed a statistically significant difference with all the variables explored. Male donors 136 (7.9%), donors age > 45 years 40 (15.3%), donors with no formal education 74 (21.5%), rural dwellers 83 (13.7%), and daily workers 101 (14.8%) showed comparatively higher seropositivity.

**Table 3:** Prevalence of serologic markers for specific TTIs and relationship to demographic factors

Socio demographic Variable	Number of Donors	Any TTI No (%)	HBsAg + ve No (%)	HCV + ve No (%)	HIV + ve No (%)	Anti –TP + No (%)
Sex						
Male	1711	234 (13.7%)	86 (5.0%)	12 (0.7%)	16 (0.9%)	136 (7.9%)
Female	228	16 (7.0%)	11 (4.8%)	1 (0.4%)	0 (0%)	4 (1.8%)
*P* -value		0.000	0.527	0.537	0.134	0.000
Age Group						
< 18	258	22 (8.5%)	13 (5.0%)	1 (0.4%)	0 (0.0%)	8 (3.1%)
19–35	912	92 (10.1%)	37 (4.1%)	4 (0.4%)	8 (0.9%)	52 (5.7%)
36–45	507	78 (15.4%)	31 (6.1%)	4 (0.8%)	6 (1.2%)	40 (7.9%)
> 45	262	58 (22.1%)	16 (6.1%)	4 (1.5%)	2 (0.8%)	40 (15.3%)
*P* -value		0.000	0.295	0.257	0.394	0.000
Educational Level						
No Education	344	93 (27%)	21 (6.1%)	2 (0.6%)	2 (0.6%)	74 (21.5%)
Elementary School	220	41 (18.6%)	14 (6.4%)	4 (1.8%)	3 (1.4%)	21 (9.5%)
Junior	339	41 (12.1%)	19 (5.6%)	0 (0.0%)	4 (1.2%)	22 (6.5%)
High School	683	55 (8.1%)	29 (4.2%)	6 (0.9%)	6 (0.9%)	19 (2.8%)
Tertiary	353	20 (5.7%)	14 (4.0%)	1 (0.3%)	1 (0.3%)	4 (1.1%)
*P* -value		0.000	0.460	0.095	0.585	0.000
Residence						
Urban	1335	129 (9.7%)	64 (4.8%)	8 (0.6%)	6 (0.4%)	57 (4.3%)
Rural	604	121 (20%)	33 (5.5%)	5 (0.8%)	10 (1.7%)	83 (13.7%)
*P*-value		0.000	0.300	0.380	0.009	0.000
Occupation						
Office worker	432	28 (6.5%)	17 (3.9%)	1 (0.2%)	1 (0.2%)	10 (2.3%)
Housewife	68	8 (11.8%)	5 (7.4%)	0 (0.0%)	0 (0.0%)	3 (4.4%)
Daily Workers	684	146 (21.3%)	41 (6.0%)	7 (1.0%)	10 (1.5%)	101 (14.8%)
Students	399	28 (7%)	18 (4.5%)	3 (0.8%)	1 (0.3%)	8 (2.0%)
Unemployed	17	3 (17.6%)	0 (0.0%)	1 (5.9%)	1 (5.9%)	1 (5.9%)
Others	339	37 (10.9%)	16 (4.7%)	1 (0.3%)	3 (0.9%)	17 (5.0%)
*P* value		0.000	0.494	0.058	0.026	0.000

Note: *P*-value, Fisher’s exact test to test statistical difference only for 2 × 2 tables, *+ ve* positive, *Any TTI* Blood unit’s positive for at least one of the tested transfusion transmissible infections

### Seropositivity rate by type of donor

The overall seroprevalence of TTIs was significantly higher (*P*-value = 0.000) in FRBD (16.8% (194/1158)) compared with VNRBD (7.2% (56/781)). In particular, the prevalence of HIV among FRBD was significantly higher compared with VNRBD (1.3% vs 0.1%; (*P*-value = 0.003). Similarly, the prevalence of syphilis among FRBD was significantly higher compared with VNRBD (10.6% vs 2.2%; *P*-value = 0.000). (Table 4).

**Table 4:** Seroprevalence of TTIs among Replacement and Voluntary Blood donors at Gash Barka Zonal Blood Transfusion Center, Barentu, from July 2014 to April 2017

Type of Donor	Number of Donors	Any TTI No (%)	HBsAg + ve No (%)	HCV + ve No (%)	HIV + ve No (%)	Anti -TP + ve No (%)
FRBD	1158	194 (16.8%)	61 (5.3%)	8 (0.7%)	15 (1.3%)	123 (10.6%)
VNRBD	781	56 (7.2%)	36 (4.6%)	5 (0.6%)	1 (0.1%)	17 (2.2%)
*P* -value		0.000	0.294	0.566	0.003	0.000

Note: *P*-value, Fisher’s exact test to test statistical difference

*FRBD* family replacement blood donors, *VNRBD* voluntary non-remunerated blood donors, *+ ve* positive, *Any TTI* Blood units positive for at least one of the tested transfusion transmissible infections

### Seropositivity and associated factors of infection

In the multivariable binary logistic regression analysis, there was a statistically significant association between HBV seropositivity and age (AOR = 1.021: 95% CI: 1.00–1.04). Similarly, the likelihood of syphilis seropositivity was higher in males (AOR = 2.63, 95% CI; 0.92–7.57) compared to females. A higher likelihood of syphilis was observed in donors with no formal education (AOR = 15.5: 95% CI: 5.5–44), elementary school (AOR = 6.0: 95% CI; 2.0–18.30), and junior level (AOR = 5.1: 95% CI; 1.73–15.13). Similarly, the likelihood of syphilis seropositivity was higher in FRBD (AOR = 1.96: 95% CI; 1.09–3.53).

A model was also fitted for the factors associated with seropositivity for any of the tested TTI. The seropositivity for any TTI was associated with age (AOR = 1.02; 95% CI: 1.01–1.04); level of education: no formal education (AOR = 4.4; 95% CI: 2.58–7.49), elementary school (AOR = 2.67; 95% CI: 1.49–4.80), and Junior school (AOR = 2.00; 95% CI: 1.14–3.52). In addition, blood from FRBD had an increased likelihood of contamination with at least one TTI (AOR =1.56; 95% CI: 1.10–2.21). Table 5 presents the odds ratio at 95% CI for HBV, Syphilis and any TTI. HIV and HCV were not modeled because of small sample size.

**Table 5:** Multivariable logistic regression model of factors associated with, HBV, Syphilis and any TTI

Variables	HBV AOR (95%CI)	Syphilis AOR (95%CI)	Any TTI AOR (95%CI)
Sex			
Female		1	
Male		2.63 (0.92–7.57)	
Age	1.021 (1.00–1.04)	1.03 (1.01–1.045)	1.02 (1.01–1.04)
Level of Education			
Tertiary Level		1	1
No Formal Education		15.5 (5.5–44)	4.4 (2.58–7.49)
Elementary School		6.0 (2.0–18.30)	2.67 (1.49–4.80)
Junior Level		5.1 (1.73–15.13)	2.00 (1.14–3.52)
High School		2.9 (1.0–8.64)^*^	1.63 (0.95–2.78)^*^
Type of Donor			
VNRBD		1	1
FRBD		1.96 (1.09–3.53)	1.56 (1.10–2.21)

*FRBD* Family Replacement Blood Donors, *VNRBD* Voluntary non-remunerated Blood Donors, *HBV* Hepatitis B virus, *AOR* Adjusted Odds Ratio, *Any TTI* Blood units positive for at least one of the tested transfusion transmissible infections

*P < 0.05

## Discussion

The rapid improvement of health care and the increased number of attendants for surgical treatment due to scaling up of surgical technology have increased the clinical demand for blood products across SSA. However, countries within the region consistently face regional and/or national shortages of blood products [10]. According to a recent WHO global report, the annual supply of blood units in Africa has consistently fallen short of the recommended threshold of 10 units/1000 people [1]. This report reinforces the findings of a previous WHO report on blood donations in SSA which noted that these donations are less than 4 units per 1000 people [1]. More importantly, systematic screening of blood units for all TTIs is not undertaken in several countries [1]. Therefore, blood safety is still a problem [2]. To minimise safety risks, donor profiling and risk assessment for specific TTIs premised on the association between demographic and behavioural variables have been promoted [16]. The fundamental need for situational analysis which clarifies geographical variation in TTI burden and/or risk has also been emphasised [10]. In the present study, the majority of the blood donors were males (88.2%). This finding was similar to the previous reports from Mozambique (89.7%) [9], Iran (90%) [16], and Burkina Faso (75.62%) [17]. The predominance of male donors could partially be linked to physiological differences between men and women, and cultural misconception of the society. Pregnancy, breastfeeding, and higher prevalence of iron deficiency anemia among women, and the popular perception that men are healthier than women may increase the number of male blood donors [10]. Similarly, a significant proportion (35.2%) of the blood donors had secondary educational level. This finding can be rationalized as an outcome of the existing policy by the blood bank which targets secondary school students as blood donors. Overall, the findings of our study conforms to the previous reports, which suggested that SSA donor pool is predominantly young and disproportionately male [10]. Broadening donor demographics through bolstering the number of women and older donors’ is of interest to increase donor numbers.

Blood donor selection strategy founded on deferral of high-risk prospective donors remains the principle line of defense against TTIs. Transfusion risk can be minimised by favouring VNRBD to FRBD, and WHO recommends that VNRBD should comprise 80% of donors [18]. The existing evidence from a survey on the status of blood safety in the WHO African region reported that 50% or more of the WHO Africa region countries are dependent on FRBD [19]. Importantly, Eritrea has not yet adopted the policy of shifting donations from FRBD to VNRBD. In the present study, the proportion of VNRBD was comparatively low. Altogether, the low proportion of VNRBD in SSA has been attributed to the fact that the recruitment of these donors is costly and logistically complex since it requires strategized recruitment, marketing and well-timed collection [10].

The overall prevalence of TTIs among blood donors was 13.7%. The frequency of TTI reported in this study was higher than the previously reported figure (3.8%) [15]. This increase in the prevalence of TTI could be attributed to the relatively higher cumulative proportion of HBV (5%) and syphilis (7.2%) markers in this region. The frequency of TTI was associated with donor type, donor’s level of education and age. This is consistent with previous studies which have reported a higher frequency of TTI among FRBD and older donors [19, 20]. The findings highlight the need to focus on empowering health education of the population, organising donor profiling, and assessing disparity in TTI burden and its associated factors to decrease the risk of TTIs.

The frequency of HBV in our study is slightly lower compared with studies conducted in parts of SSA [5, 20, 21]. In the present study, the lower risk for transfusion transmissible HBV could be explained by the excessive use of rapid diagnostic technique (HBsAg Kit), which may compromise the case detection rate as the virus has an acute window period. Over-reliance on HBsAg kits may underestimate transmission risk compared with hepatitis B core antigen (Anti-HBc) [21].

In the multivariate model, the risk of HBV increased as age increases (modeled as a continuous variable). However, the HBV prevalence was similar between VNRBD and FRBD. Similarly, there was no any relationship between HBV and other TTIs, suggesting that the epidemiology of HBV is not necessarily linked to that of HIV, HCV or syphilis. This finding was different from the previous reports [21–23], which could be due to behavioural, socio-cultural and socio-economic differences. Therefore, it is of importance to understand the mode of transmission of HBV in this population.

The prevalence of HCV markers in this study was higher (0.7%) than the previous national average (0.18%) [15]. However, the reported prevalence of HCV was comparatively low compared with previous studies in Nigeria (1.4%) [24], Ethiopia (3.5%) [21], and Equatorial Guinea (3.71%) [25]. We observed no statistically significant difference between VNRBD and FRBD in HCV prevalence. This finding may be attributed to the low proportion of HCV seropositive cases. To speculate, iatrogenic transmission by unsafe injections - a mode of transmission that has been identified as the primary mechanism for new and sustained infection in some parts of Africa, should be a viable area of interest [10].

In general, the comparatively high seroprevalence of anti-Treponemal antibodies highlighted in the foregoing paragraph is suggestive of a high-density carrier state or active infection. The frequency of syphilis was comparatively higher among donors of the age category > 45 years, (15.3%) followed by 36–45 aged donors (7.9%), consistent with a previous study [26]. The study conducted in Israel has reported that donors in the age category between 35 and 44 years-old and older than 45 years-old had an increase of 6.5 and 7.4 folds in the prevalence of syphilis, respectively, compared with the younger donors (aged 24 years or less) [26]. In the present study, males had a higher frequency of syphilis compared with females. This is consistent with a study conducted in Tanzania which reported an increased preponderance of syphilis in males (4.8%) than females (4.0%) [27]. The proportion of syphilis was higher among daily workers (14.8%) compared with students (2%). Also, the prevalence of syphilis was significantly associated with occupation and donor type. In addition, a higher level of education was associated with a reduced likelihood of having syphilis in this study.

In the present study, the overall seroprevalence for HIV was 0.8%. The seroprevalence for HIV estimate is less than the value reported in the previous study [15]. Importantly, a marked difference in HIV prevalence was observed among blood donors. The seroprvalence for HIV was higher among individuals with elementary educational level, daily workers, and FRBD donors. The findings highlight the need of comprehensive and targeted health education to enhance the awareness of the community on the risk and mode of transmission of HIV.

### Limitations

The major limitation of this study resides in the fact that the actual burden of TTIs such as HIV, HBV and HCV could have been underestimated due to the presence of a window period. In addition, this study does not consider the range of risk factors associated with TTIs, which may limit the scope of the study. A comprehensive study evaluating an expanded range of risk factors would have provided an insight into TTI prevention from a local standpoint. Irrespective of the highlighted limitations and the fact that this study has a largely local relevance; it’s our position that due to the ongoing global migration, the study may provide useful information to blood services worldwide.

## Conclusions

The present study clearly shows a high seroprevalence of TTIs among blood donors at Gash Barka Zonal Blood Transfusion Center, Barentu. Syphilis and HBV had the highest prevalence followed by HIV and HCV. The preference for VNRBD over FRBD is not necessarily justifiable. Although inter-regional comparisons demonstrated that these frequencies are generally low in most part, the situation suggests a possible increase in TTI overtime. Therefore, epidemiological research focused on the identification of risk factors and well-targeted sensitization campaigns should be prioritized. The need to develop a well validated donor questionnaire adapted to the local epidemiologic and social characteristics of the region should be explored.

## Abbreviations

Anti-HBc: Anti Hepatitis B core antigen
Anti-TP: Anti *Treponema pallidum* antibodies
CI: Confidence Interval
CMV: Cytomegalovirus
EIA: Enzyme Immuno Assays
ELISA: Enzyme Linked Immune Sorbent Assay
FRBD: Family Replacement Blood Donors
HBsAg: Hepatitis B surface Antigen
HBV: Hepatitis B Virus
HCV: Hepatitis C Virus
HIV: Human Immunodeficiency Virus
HTLV: Human T Lymphotropic Virus
NAT: Nucleic Acid Testing
NBTC: National Blood Transfusion Center
OR: Odds Ratio
SSA: Sub-Saharan Africa
STI: Sexually Transmitted Infections
TTIs: Transfusion Transmitted Infections
VNRBD: Voluntary Non-Remunerated Blood Donors
WHO: World Health Organization
